# Supplementary material for: Slight compositional variation-induced structural disorder-to-order transition enables fast Na+ storage in layered transition metal oxides
Source: Nat Commun. 2022 Dec 22;13:7888. doi: 10.1038/s41467-022-35597-4 (PMC9780345; doi:10.1038/s41467-022-35597-4)
Supplement: Supplementary file 1 — Supplementary Information File [file 41467_2022_35597_MOESM1_ESM.pdf]

Supplementary Information

Slight compositional variation-induced structural disorder-to-order transition enables fast Na^+ storage in layered transition metal oxides

Yuansheng Shi[1], Pengfeng Jiang[1], Shicheng Wang[1], Weixin Chen[1], Bin Wei[1], Xueyi Lu[1], Guoyu Qian[1], Wang Hay Kan[2,3], Huaican Chen[2,3], Wen Yin[2,3], Yang Sun[1], and Xia Lu[1,]*

1. School of Materials, Sun Yat-sen University, Shenzhen, 518107, P. R. China.

2. Spallation Neutron Source Science Center, Dongguan, 523803, P. R. China.

3. Institute of High Energy Physics, Chinese Academy of Sciences, Beijing, 100049, P. R. China.

[*]E-mail: luxia3@mail.sysu.edu.cn

Supplementary Figures

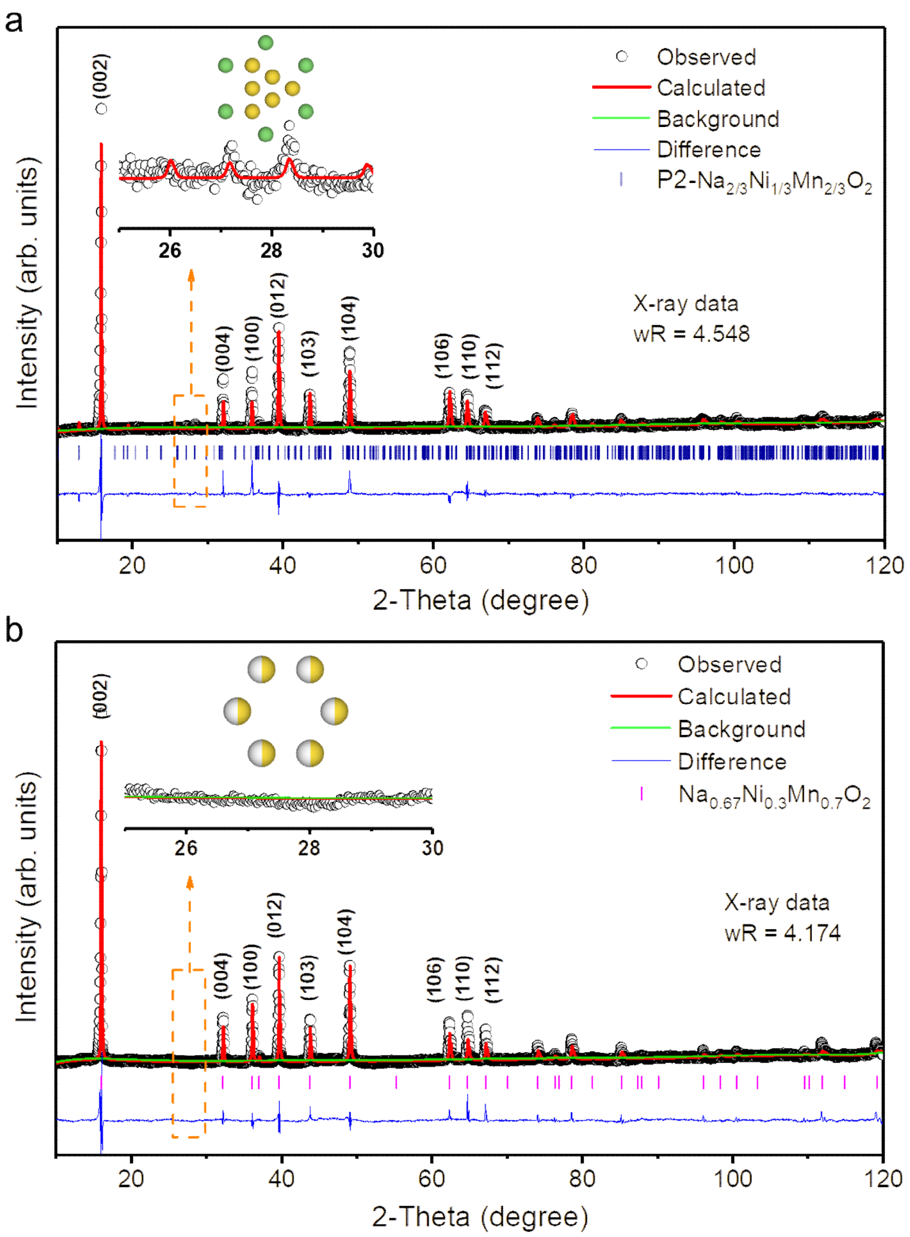

Figure S1. Refined X-ray powder diffraction pattern for (a) P2-$Na_{2/3}Ni_{1/3}Mn_{2/3}O_2$ and (b) P2-$Na_{2/3}Ni_{0.3}Mn_{0.7}O_2$. wherein the experimental (black circles) and calculated (red solid line), the Bragg reflection peaks (purple and magenta solid ticks) and the difference curve (blue line) are shown, respectively. The green ball represents Na_f and the yellow ball stands for Na_e.

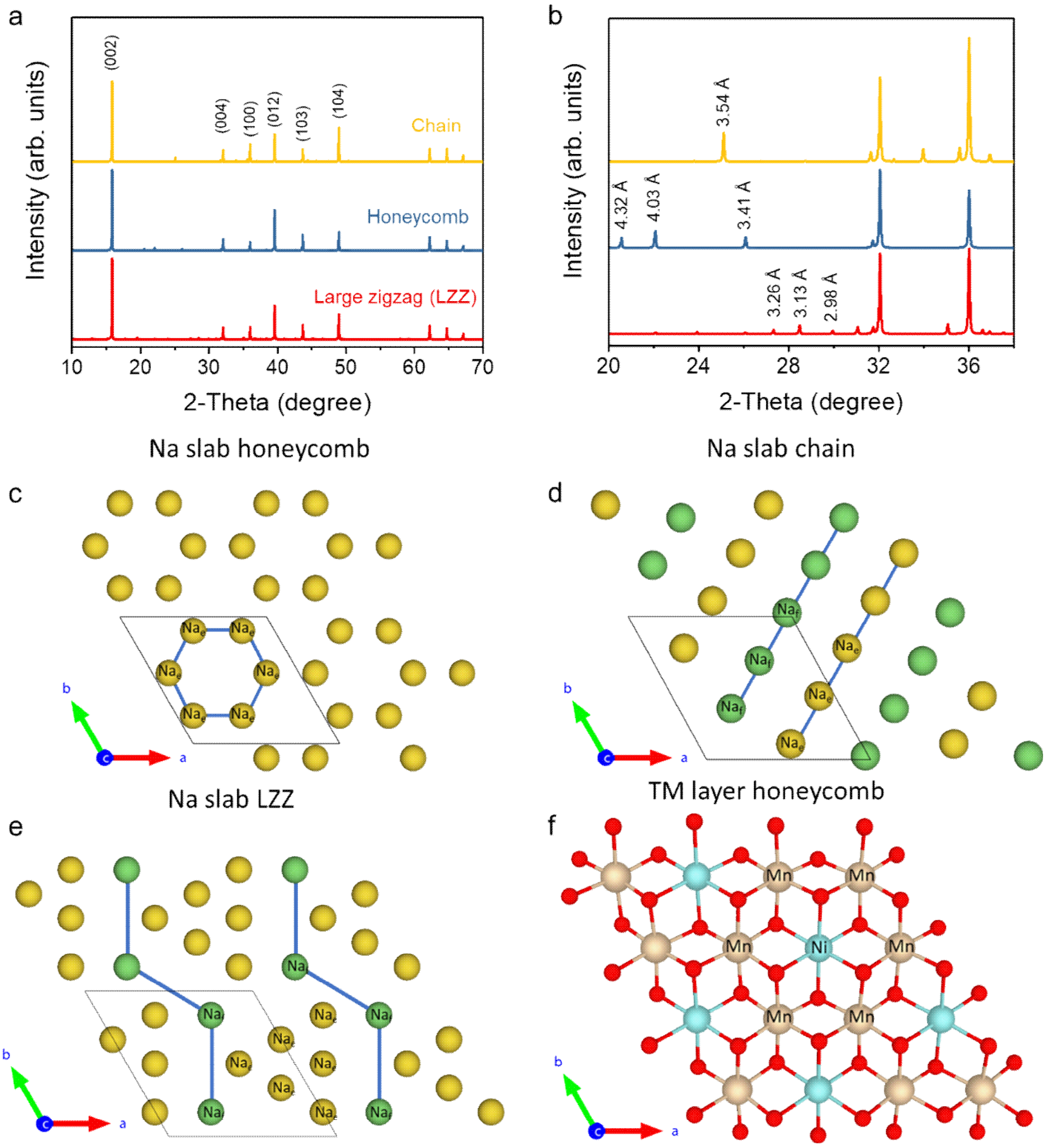

Figure S2. (a) Simulated XRD patterns of P2-type layered oxides with three different in-plane Na^{+}-ion/vacancy ordering (large zigzag, honeycomb and chain type). (b) A magnified view of XRD patterns between 20-38°, where the d-spacings correspond to the average distances between the adjacent intralayer sodium ions in the in-plane Na^{+}-ion/vacancy ordering arrangements. The atomic structures of P2-$Na_{2/3}Ni_{1/3}Mn_{2/3}O_2$ with three different in-plane Na^{+}-ion/vacancy orderings, (c) honeycomb type, [occ (Na_f) = 0]; (d) chain type, [occ (Na_f) = 1/2] and (e) large zigzag (LZZ), [occ (Na_f) = 1/6]. The green ball represents the Na_f site and the yellow ball stands for Na_e site. (f) The atomic arrangements of Mn^{4+} and Ni^{2+} ions described by honeycomb ordering in the transition metal planes of P2-$Na_{2/3}Ni_{1/3}Mn_{2/3}O_2$.

Supplementary Note 1. The XRD/NPD data analysis is based on crystallography. The phenomenal peaks are the direct responses to the spatial ordering arrangement of one or more species of atoms in one specific crystal structure. In terms of superstructures, they always respond with small intensities at lower diffraction angles, where they have larger lattice constants than the original structure. Hence, the high energy XRD, or NPD, is generally required to characterize these small or minor peaks.

In detail, three types of in-plane Na^+-ion/vacancy orderings can be found in P2 phase according to the structural enumeration upon DFT simulations. Figure S2 shows the simulated XRD patterns of the above ordering structures using RIETAN-FP. Owing to the similar X-ray scattering factor of Ni and Mn ions, they can not be distinguished clearly. While, the small superlattice peaks in X-ray diffraction patterns can be assigned to the in-plane Na^+-ion/vacancy orderings, corresponding to the d-spacings of the adjacent intralayer sodium ions. In Figure 1 of the manuscript, the clear LZZ superstructure peaks are found at 27.3° and 28.4° for P2-$Na_{2/3}Ni_{1/3}Mn_{2/3}O_2$ (inset of Figure 1a), corresponding to the d-spacings of 3.13 and 3.26 Å, respectively, which are absent in the $Na_{2/3}Ni_{0.3}Mn_{0.7}O_2$ pattern to show the structural differences.

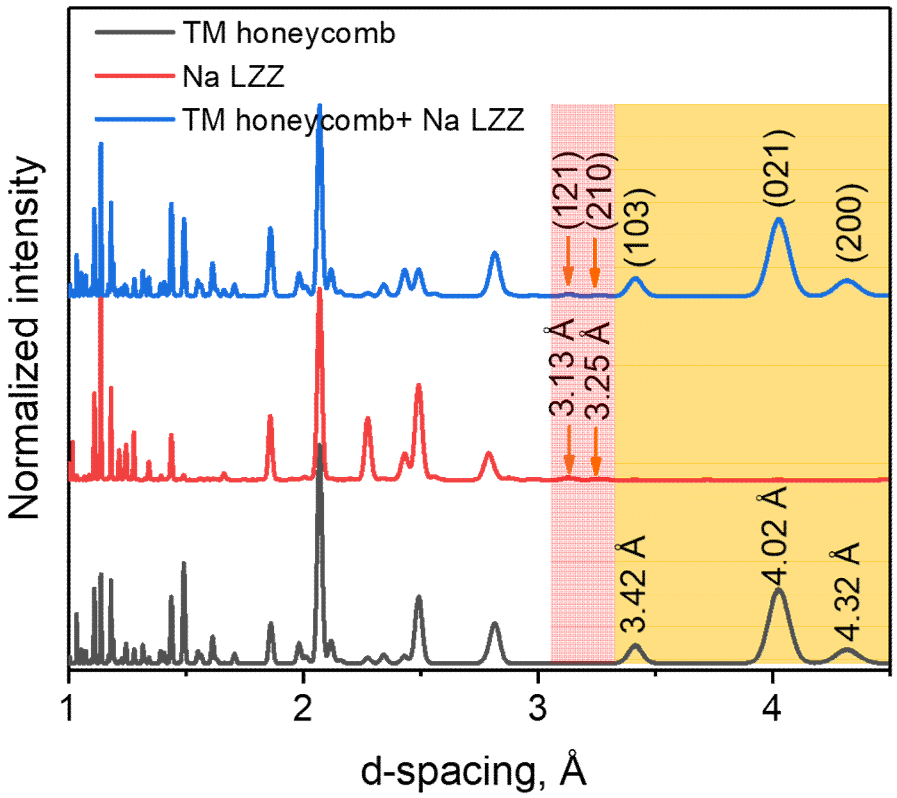

Figure S3. Simulated NPD data of P2-type layered oxides with the transition metal (TM) honeycomb ordering, the Na slab large zigzag(LZZ) ordering and the coexistence of TM honeycomb and Na LZZ ordering using GSAS II. The superstructure peaks (TM honeycomb ordering and Na slab LZZ ordering) were clearly indexed based on the provided crystal structures in table S1 with space Group of *P*3.

Supplementary Note 2. As for the neutron powder diffraction (NPD), the strong neutron scattering length contrasts of Ni (10.3 fm) and Mn (-3.73 fm) enable it to be an effective measurement to detect the Ni−Mn intralayer ordering as shown in Figure 1b. Figure S3 shows the NPD results' peak differences between transition metal (TM) honeycomb and Na slab large zigzag (LZZ) ordering. The d-spacings of 3.13 and 3.26 Å indicate the Na slab LZZ ordering in the red shadowed regions, which are in line with the experimental data in Figure 1b of the revised manuscript. Moreover, the simulation results point out that the d-spacings of 3.42, 4.02 and 3.26 Å can be assigned to the TM honeycomb ordering, which are shadowed with the yellow color in Figure 1b and Figure S3 in SI.

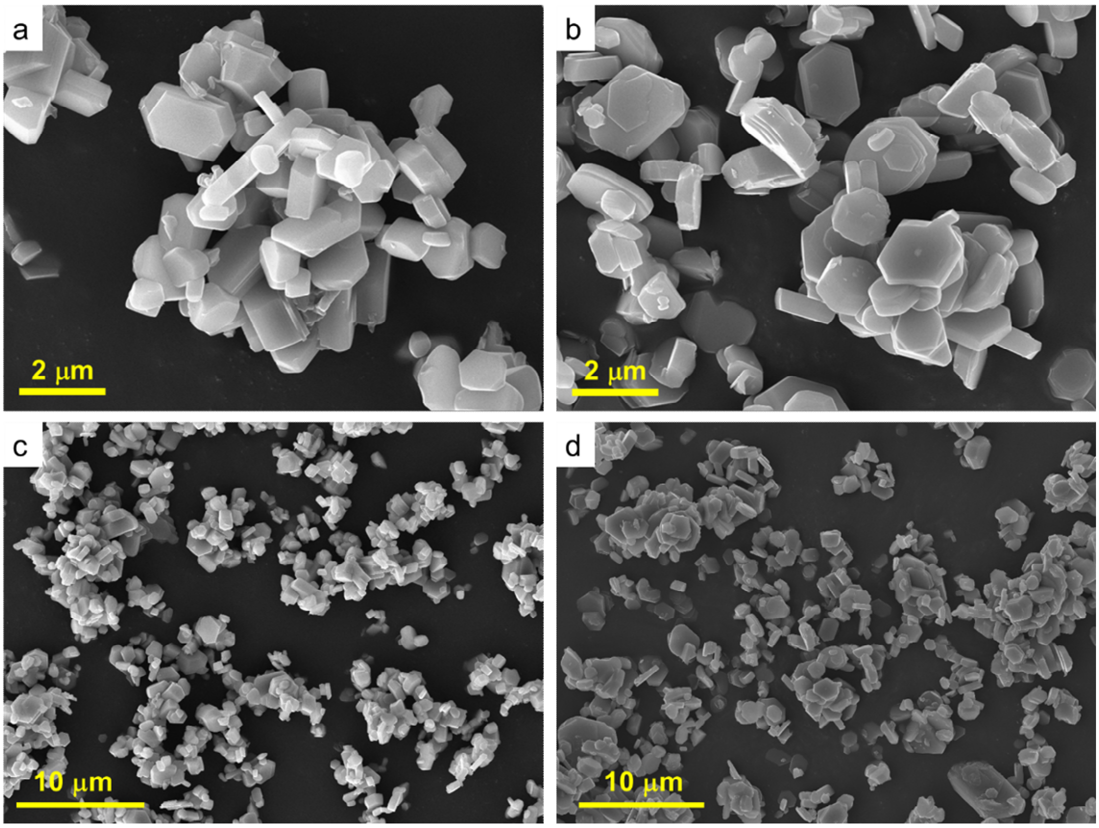

Figure S4. SEM images of (a) P2-$Na_{2/3}Ni_{1/3}Mn_{2/3}O_2$ and (b) P2-$Na_{2/3}Ni_{0.3}Mn_{0.7}O_2$ in a small area. SEM images of (c) P2-$Na_{2/3}Ni_{1/3}Mn_{2/3}O_2$ and (d) P2-$Na_{2/3}Ni_{0.30}Mn_{0.70}O_2$ at large scale view. These two samples both display the schistose particles in microsize ranging from 1-5 μm.

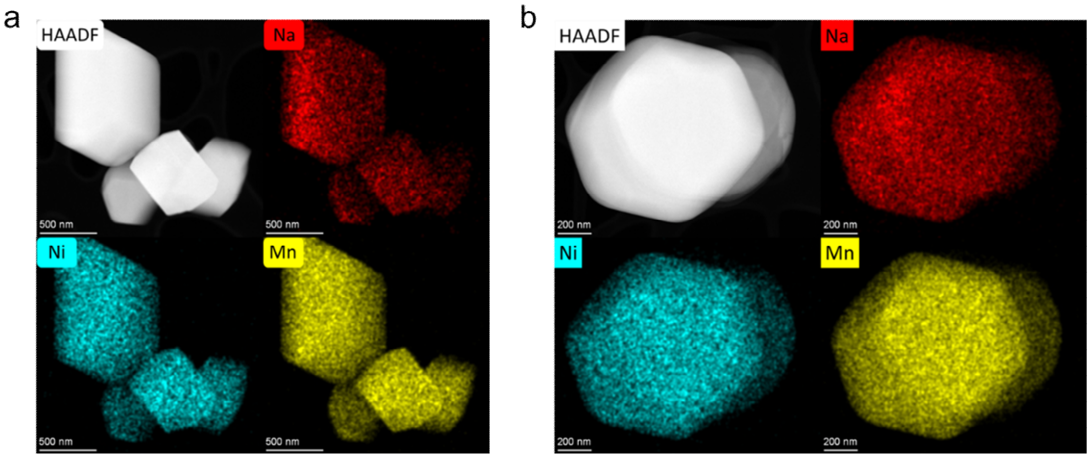

Figure S5. EDX elemental mappings of (a) P2-$Na_{2/3}Ni_{1/3}Mn_{2/3}O_2$ and (b) P2-$Na_{2/3}Ni_{0.3}Mn_{0.7}O_2$.

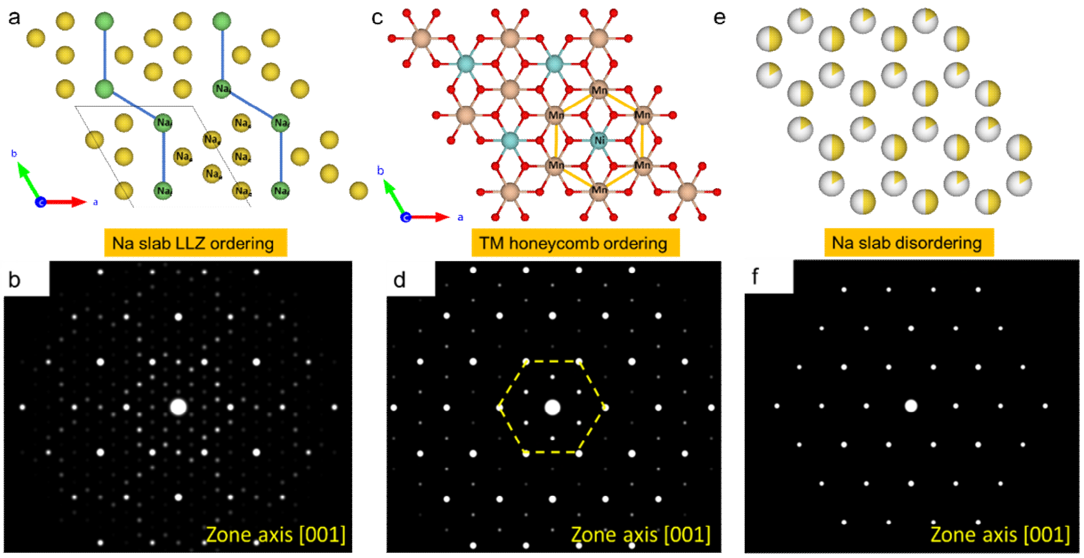

Figure S6. (a) Atomic arrangement of Na slab LLZ ordering for P2-type layered oxides, simulated SAED images of (b) Na slab LLZ ordering. (c) Atomic arrangement of honeycomb ordering in TM layer for P2-type layered oxides, and the corresponding simulated SAED images of (d) TM honeycomb ordering at [001] zone axis. (e) Atomic arrangement of Na ions disordering in Na slab for P2-type layered oxides, simulated SAED images of (f) Na ions disordering at [001] zone axis.

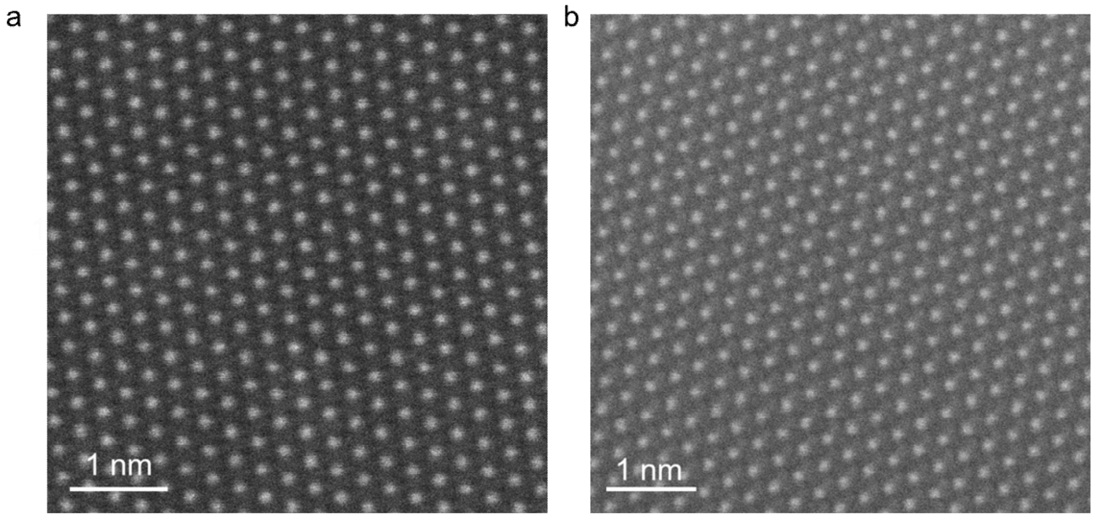

Figure S7. HAADF-STEM images of (a) P2-$Na_{2/3}Ni_{1/3}Mn_{2/3}O_2$ and (b) P2-$Na_{2/3}Ni_{0.3}Mn_{0.7}O_2$ in the [001] crystal direction.

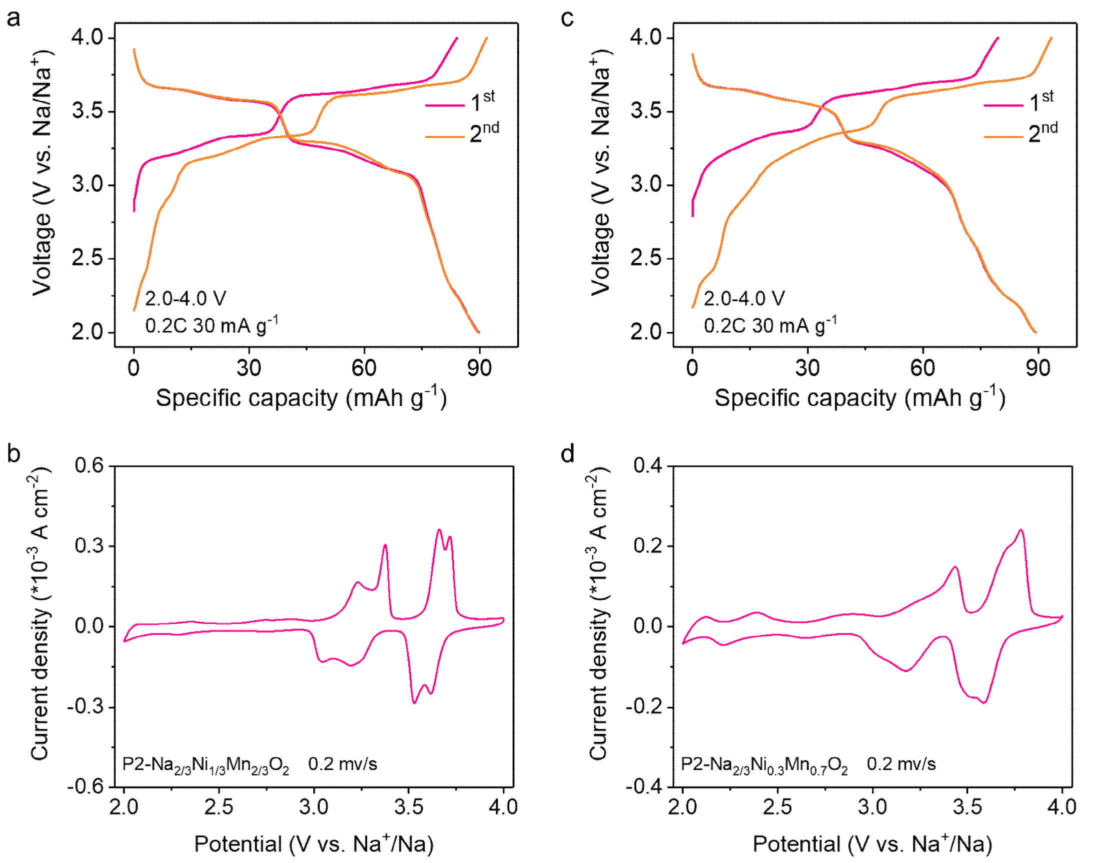

Figure S8. Charge/discharge curves of (a) P2-$Na_{2/3}Ni_{1/3}Mn_{2/3}O_2$ and (c) P2-$Na_{2/3}Ni_{0.3}Mn_{0.7}O_2$ at 0.2C (30 mA g^{-1}) for the first two cycles between 2.0 and 4.0 V. And the CV curves of (b) P2-$Na_{2/3}Ni_{1/3}Mn_{2/3}O_2$ and (d) P2-$Na_{2/3}Ni_{0.3}Mn_{0.7}O_2$ for the first cycle at 0.2 mV/s.

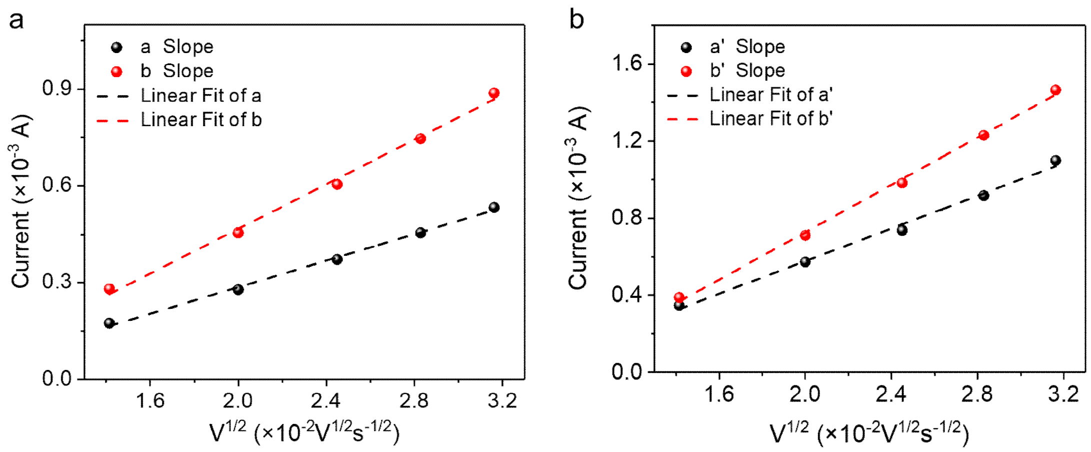

Figure S9. (a) the dependence of current of the peaks marked in Figure 2e on the square root of the scan rate ($v^{1/2}$) for P2-$Na_{2/3}Ni_{0.3}Mn_{0.7}O_2$. (b) the dependence of current of the peaks marked in Figure 2f on the square root of the scan rate ($v^{1/2}$) for P2-$Na_{2/3}Ni_{1/3}Mn_{2/3}O_2$ electrode.

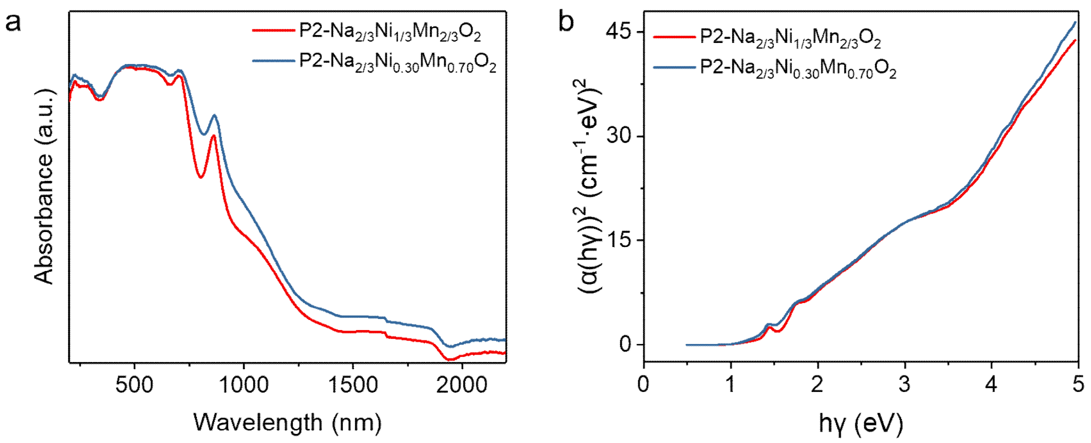

Figure S10. (a) Experimental ultra-visible light absorption spectra of P2-$Na_{2/3}Ni_{1/3}Mn_{2/3}O_2$ and P2-$Na_{2/3}Ni_{0.30}Mn_{0.70}O_2$ with the wavelength. (b) Dependence of $(\alpha h\nu)^2$ *vs.* photon energy (*hv*), from which the optic band gap is derived, above results indicate optic band gap of P2-$Na_{2/3}Ni_{1/3}Mn_{2/3}O_2$ and P2-$Na_{2/3}Ni_{0.30}Mn_{0.70}O_2$ is equivalent roughly.

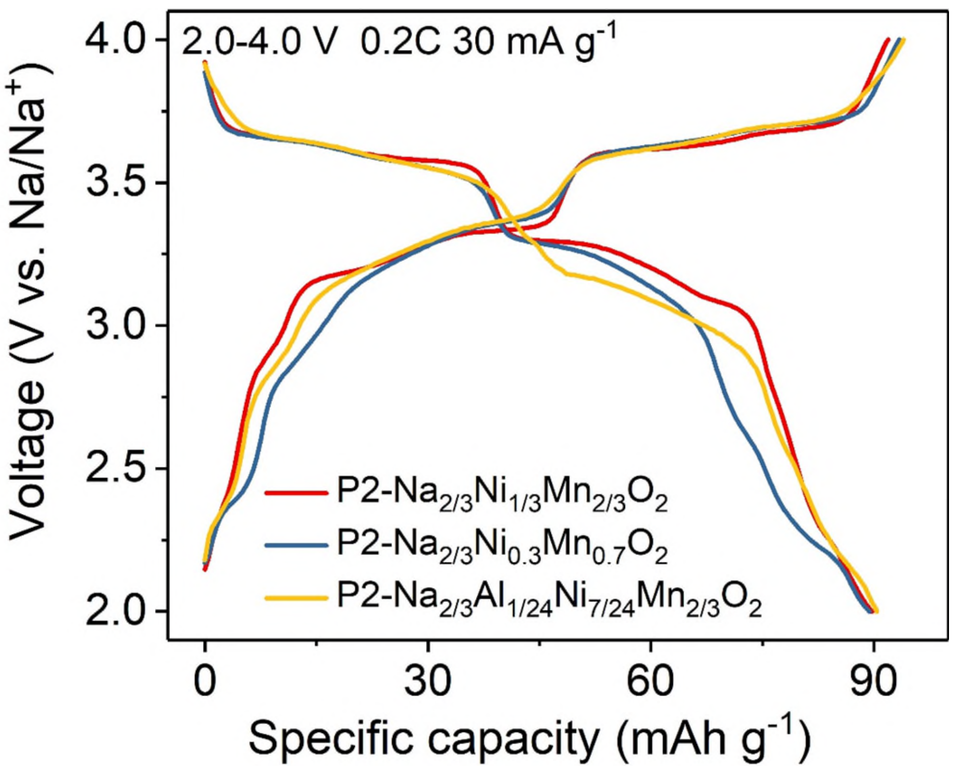

Figure S11. Charge/discharge curves of P2-$Na_{2/3}Ni_{1/3}Mn_{2/3}O_2$, P2-$Na_{2/3}Ni_{0.3}Mn_{0.7}O_2$ and P2-$Na_{2/3}Al_{1/24}Ni_{7/24}Mn_{2/3}O_2$ at 0.2C (30 mA g^{-1}) in the 2nd cycle between 2.0 and 4.0 V.

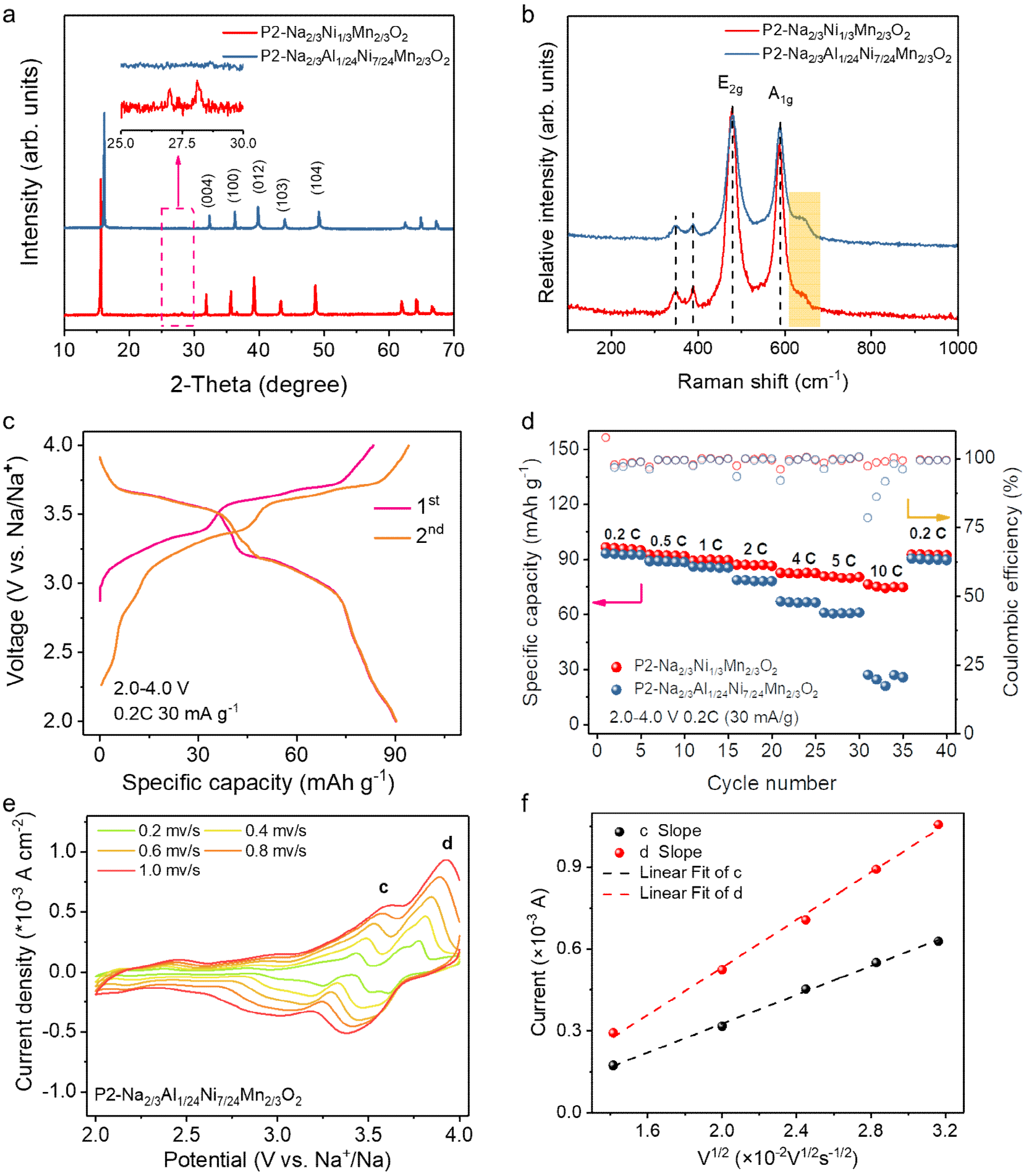

Figure S12. (a) XRD patterns of the as-prepared P2-$Na_{2/3}Ni_{1/3}Mn_{2/3}O_2$ and P2-$Na_{2/3}Al_{1/24}Ni_{7/24}Mn_{2/3}O_2$ sintered at 950℃ for 15h, the inset indicates the loss of in-plane Na^+ - ion/vacancy ordering in P2-$Na_{2/3}Al_{1/24}Ni_{7/24}Mn_{2/3}O_2$. (b) Raman spectra of P2-$Na_{2/3}Ni_{1/3}Mn_{2/3}O_2$ and P2-$Na_{2/3}Al_{1/24}Ni_{7/24}Mn_{2/3}O_2$. (c) Charge/discharge curves of P2-$Na_{2/3}Al_{1/24}Ni_{7/24}Mn_{2/3}O_2$ between 2.0 and 4.0 V at 0.2 C. (d) Rate performance. (e) CV curves of P2-$Na_{2/3}Al_{1/24}Ni_{7/24}Mn_{2/3}O_2$ electrode at different scanning rates between 2.0 - 4.0 V. (f) the dependence of current of the peaks marked in Figure S12 e on the square root of the scan rate ($v^{1/2}$) for P2- $Na_{2/3}Al_{1/24}Ni_{7/24}Mn_{2/3}O_2$ electrode.

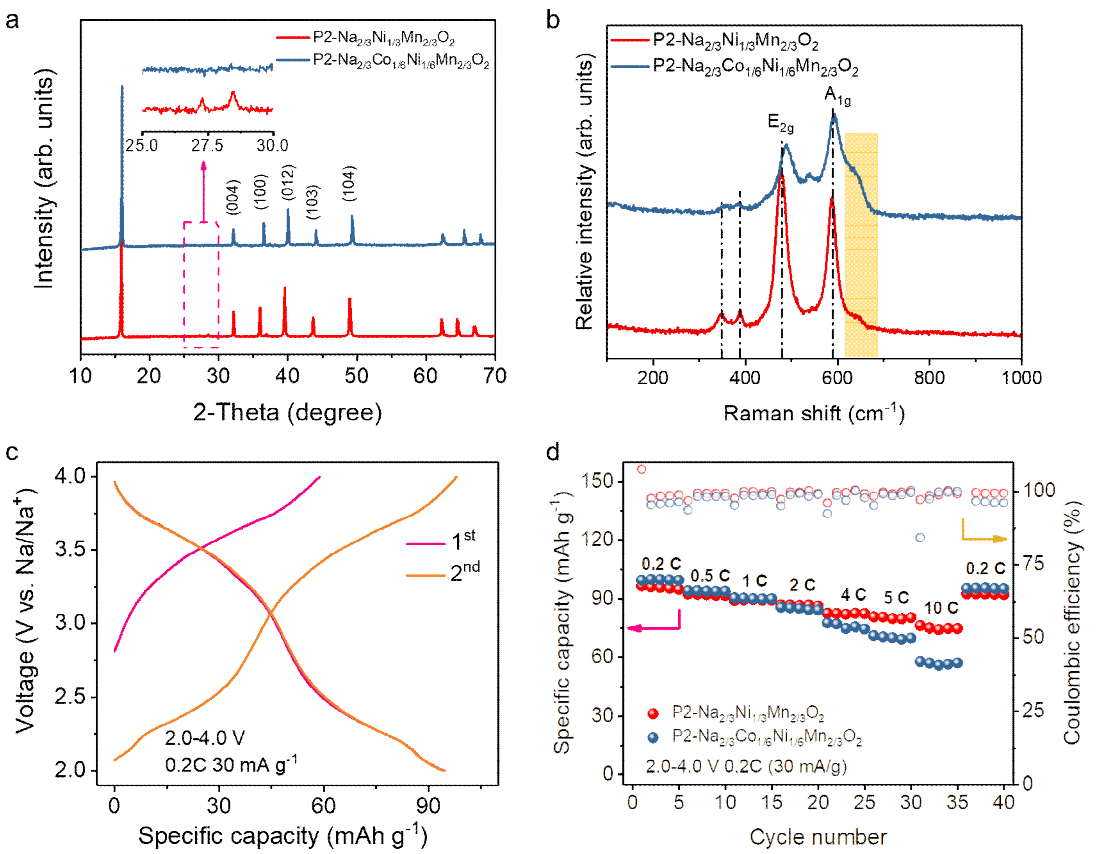

Figure S13. (a) XRD patterns of the as-prepared P2-$Na_{2/3}Ni_{1/3}Mn_{2/3}O_2$ and P2-$Na_{2/3}Co_{1/6}Ni_{1/6}Mn_{2/3}O_2$ sintered at 950℃ for 15h, the inset indicates the loss of in-plane Na^+ - ion/vacancy ordering in P2-$Na_{2/3}Co_{1/6}Ni_{1/6}Mn_{2/3}O_2$. (b) Raman spectra of P2-$Na_{2/3}Ni_{1/3}Mn_{2/3}O_2$ and P2- $Na_{2/3}Co_{1/6}Ni_{1/6}Mn_{2/3}O_2$. (c) Charge/discharge curves of P2-$Na_{2/3}Co_{1/6}Ni_{1/6}Mn_{2/3}O_2$ between 2.0 and 4.0 V at 0.2 C. (d) Rate performance.

Supplementary Note 3. The P2-$Na_{2/3}Al_{1/24}Ni_{7/24}Mn_{2/3}O_2$ and P2-$Na_{2/3}Co_{1/6}Ni_{1/6}Mn_{2/3}O_2$ samples were synthesized via a facile solid-state reaction method using the stoichiometric amounts of Na_2CO_3 (99.99%; Macklin; an excessive 2% of Na_2CO_3 is added owing to the volatilization loss), MnO_2 (99.9 %; Alfa Aesar), NiO (99.5%; Aladdin), Al_2O_3 (99.9%; Aladdin) or Co_2O_3 (AR; Macklin) as precursors. The mixed powders were ball milled for 24 hours. Then, the powders were preheated in a muffle furnace at 450 °C for 6 h followed by annealing at 950 °C for 15 h. The products were cooled naturally down to room temperature, then ground and transferred to an argon-filled glovebox for protection.

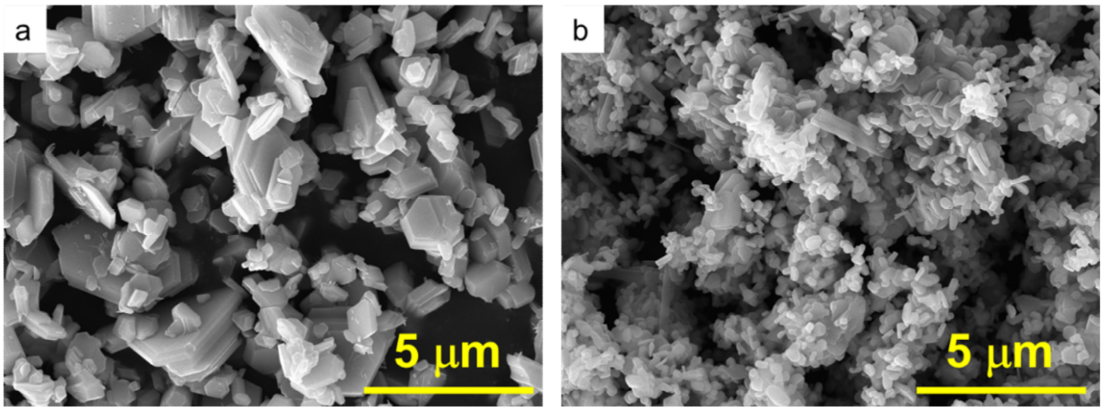

Figure S14. (a) SEM images of (a) P2-$Na_{2/3}Ni_{1/3}Mn_{2/3}O_2$ and (b) P3- $Na_{2/3}Ni_{1/3}Mn_{2/3}O_2$.

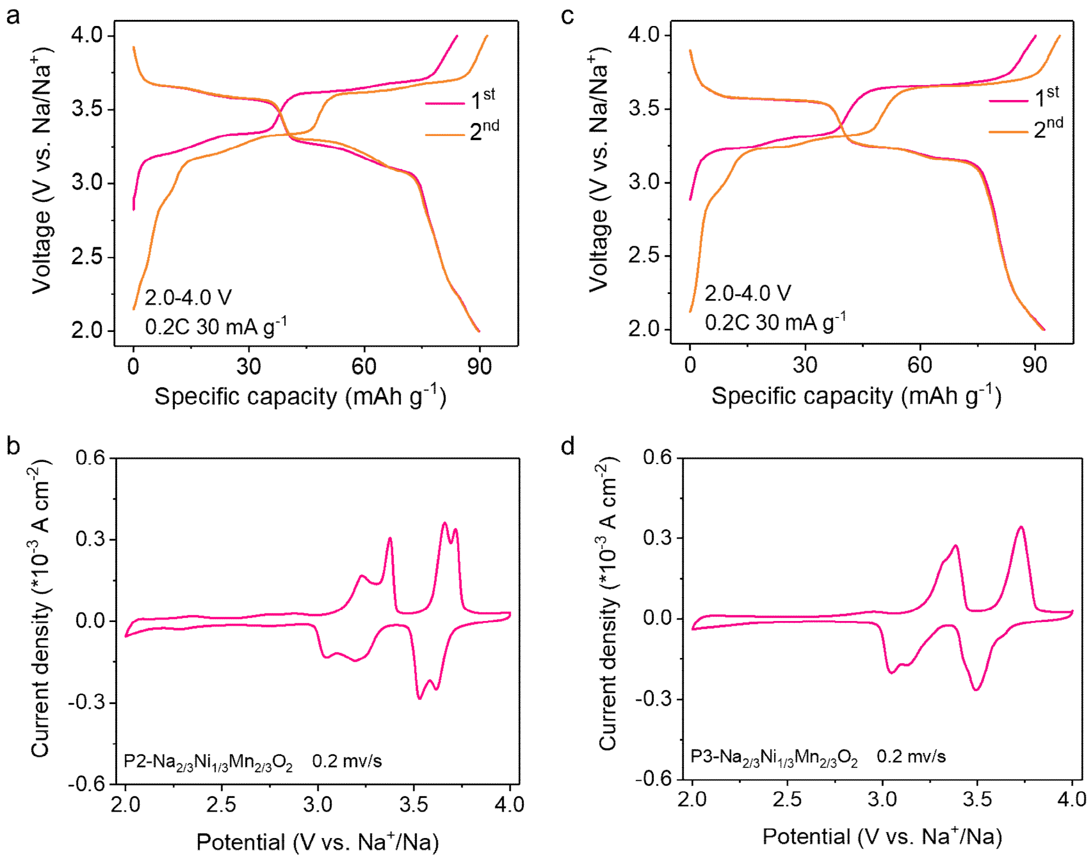

Figure S15. Charge/discharge curves of (a) P2-$Na_{2/3}Ni_{1/3}Mn_{2/3}O_2$ and (c) P3-$Na_{2/3}Ni_{1/3}Mn_{2/3}O_2$ at 0.2C (30 mA g^{-1}) for the first two cycles between 2.0 and 4.0 V. And the CV curves of (b) P2-$Na_{2/3}Ni_{1/3}Mn_{2/3}O_2$ and (d) P3- $Na_{2/3}Ni_{1/3}Mn_{2/3}O_2$ for the first cycle at 0.2 mV/s.

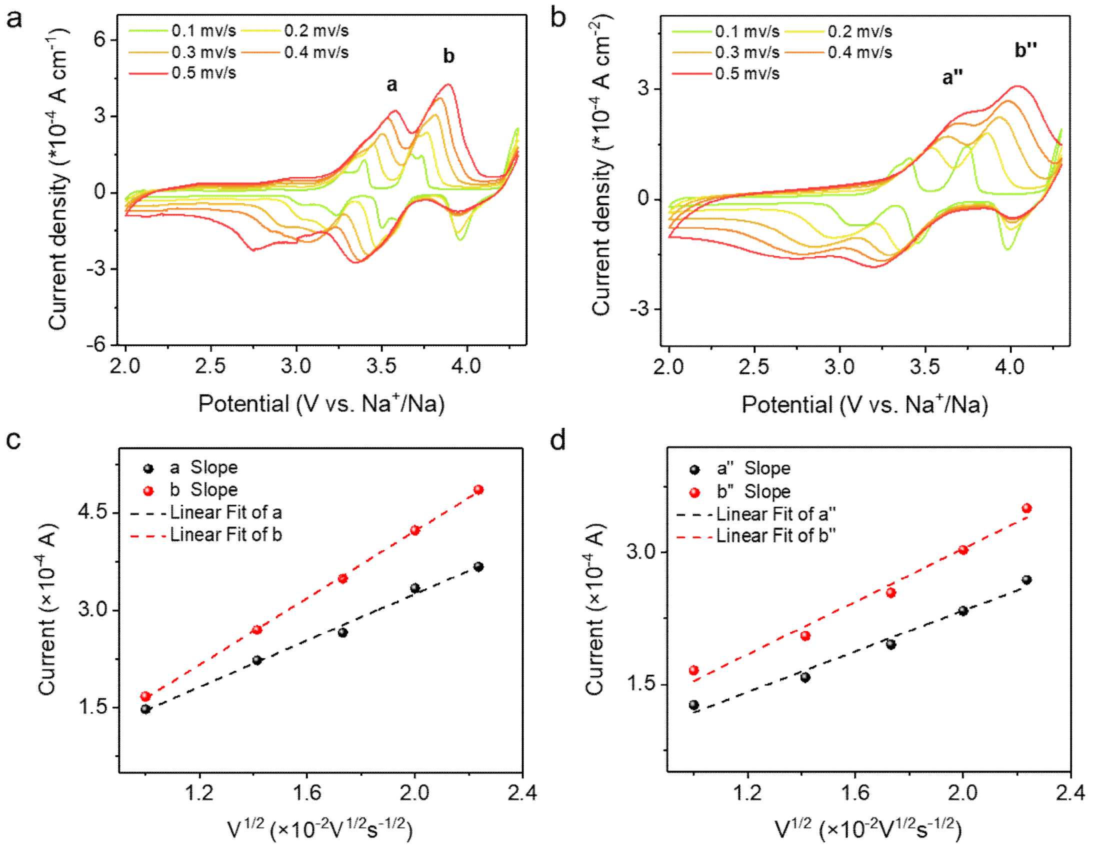

Figure S16. CV curves of (a) P2-$Na_{2/3}Ni_{1/3}Mn_{2/3}O_2$ (b) and P3-$Na_{2/3}Ni_{1/3}Mn_{2/3}O_2$ electrode at different scan rates (0.1, 0.2, 0.3, 0.4, 0.5 unit mV s^{-1}) between 2.0-4.3 V; (g) The dependence of current of the peaks marked in Figure S16a on the square root of the scan rate ($v^{1/2}$) for P2-$Na_{2/3}Ni_{1/3}Mn_{2/3}O_2$; (i) the dependence of current of the peaks marked in Figure S16b on the square root of the scan rate ($v^{1/2}$) for pristine P3-$Na_{2/3}Ni_{1/3}Mn_{2/3}O_2$.

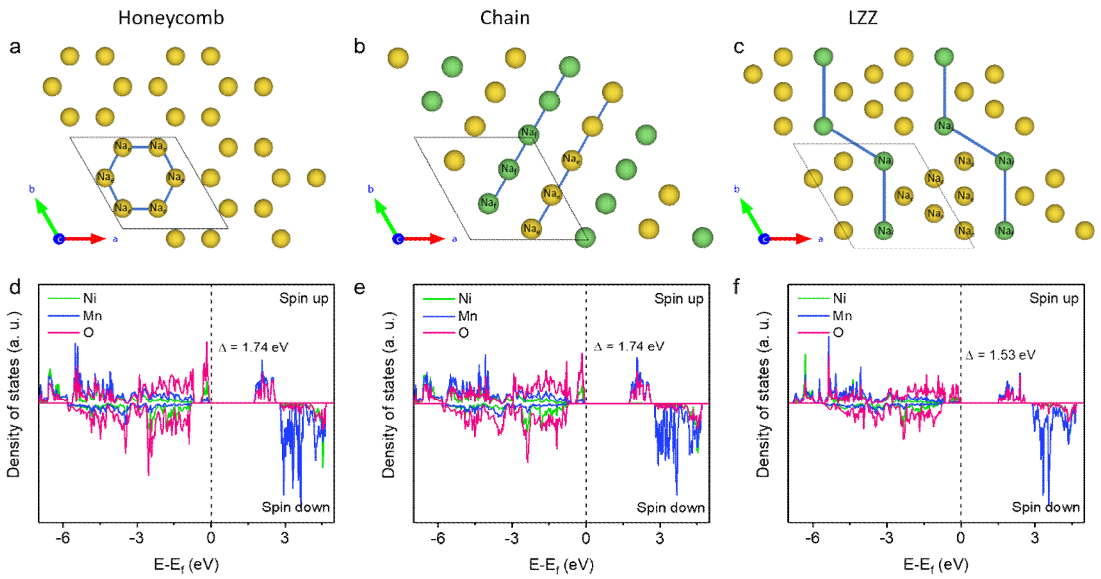

Figure S17. Atomic structure of P2-$Na_{2/3}Ni_{1/3}Mn_{2/3}O_2$ with three different in-plane Na^+-ion/vacancy orderings. (a) Honeycomb [occ (Na_f) = 0], (b) chain [occ (Na_f) = 1/2], (c) large zigzag (LZZ) [occ (Na_f) = 1/6] types. The green ball represents Na_f and the yellow ball stands for Na_e. The DOSs of P2-$Na_{2/3}Ni_{1/3}Mn_{2/3}O_2$ with the (d) honeycomb, (e) chain and (f) LZZ type orderings.

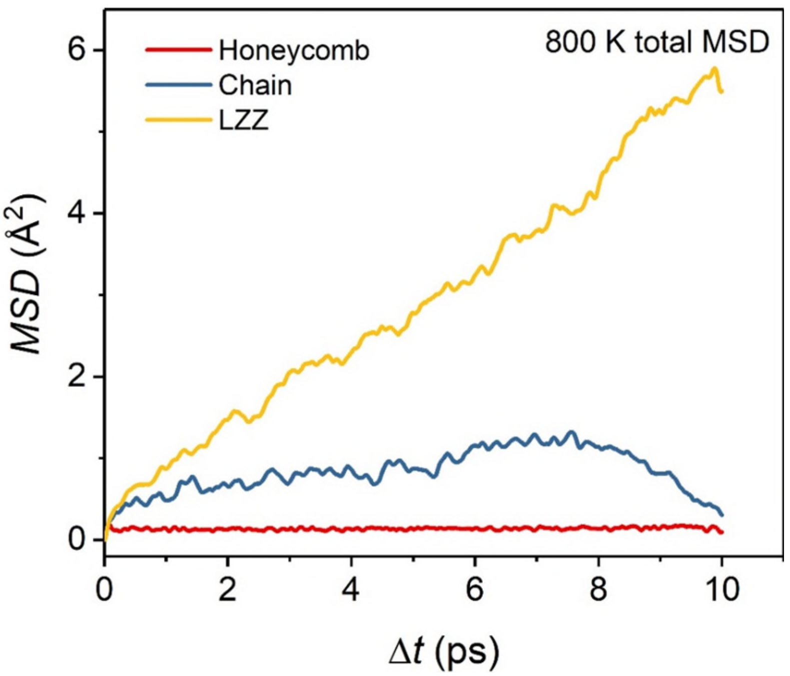

Figure S18. Total MSDs for Na-ions in P2-$Na_{2/3}CoO_2$ with different occupation way of Na at 800K. The noticeable migration of Na is captured at the Na slab with LZZ ordering. In contrast, no directional movement of Na ions is found in the other two occupation way of Na slab.

Supplementary Note 4.

Ab initio molecular dynamics (AIMD) simulations were carried out for the canonical (NVT) ensemble using a Nosé thermostat at 800 K. The cut off energy of 350 eV was setted to simulate the disordered P2-$Na_{2/3}Ni_{1/3}Mn_{2/3}O_2$ considering the current computing capabilities. The volume and the shape of the cell were fixed. The corresponding structures were heated up to the targeted temperature by the velocity scaling over 2 *ps*, and then equilibrated at the desired temperature. The timescale of the simulations was 10 *ps* and a time step of 2 *fs* was used to integrate the equation of motion. The overall disorder 1 [occ. (Na_f) = 1/6] and disorder 2 [occ. (Na_f) = 1/12] that close to expermental results was setted to obtain two randomly arranged Na configurations.

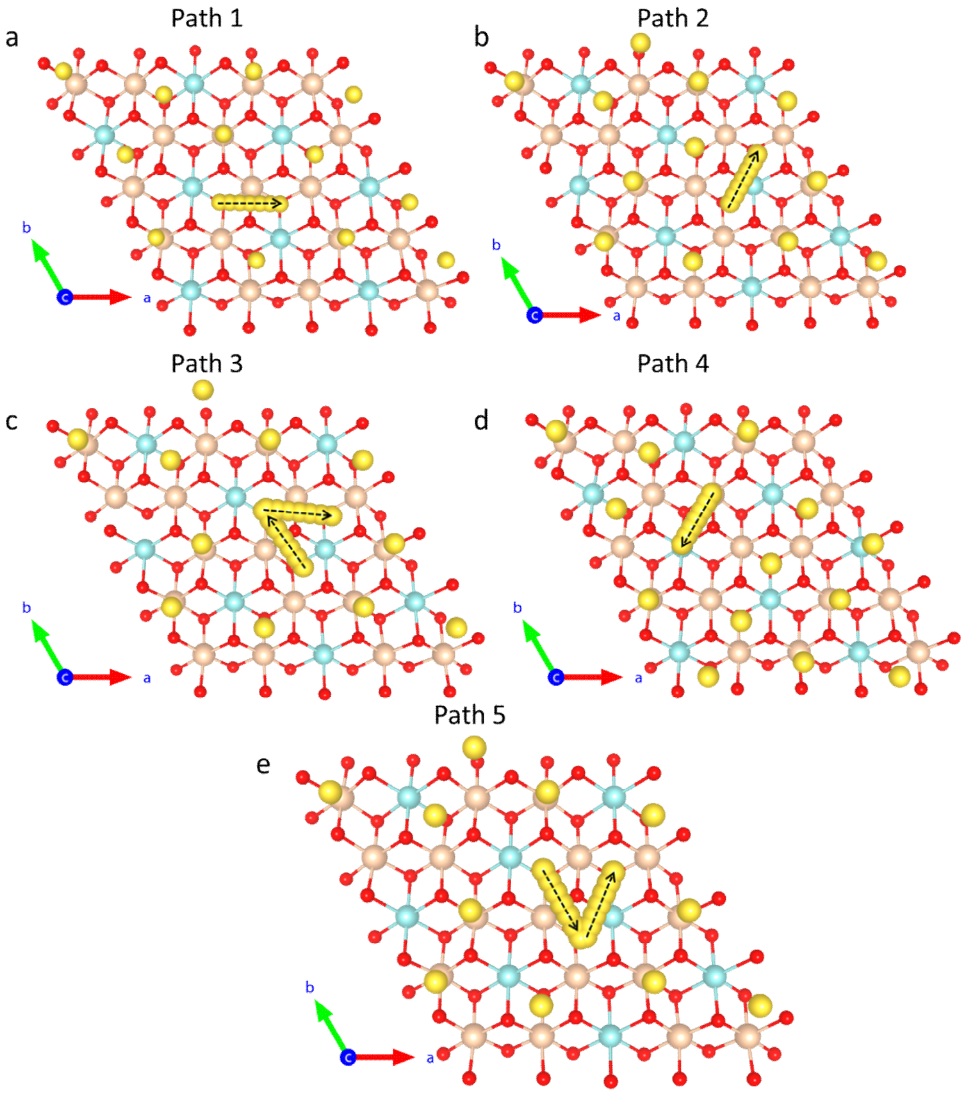

Figure S19. Na-ion diffusion in P2-$Na_{2/3}Ni_{1/3}Mn_{2/3}O_2$ of Chain type ordering ($3a \times 3b \times 1c$ supercell) with monovacancy. The illustration of (a-e) Paths 1-5 in P2- $Na_{2/3}Ni_{1/3}Mn_{2/3}O_2$ atomic structure. Path1 :the migration of one Na^+ ions within the *ab* plane, where Path 1 is closer to the Mn in TM layer. (Na_e-Na_e) Path 2: the migration of one Na^+ ions within the *ab* plane, where Path 2 is closer to the Ni in TM layer. (Na_e-Na_e) Path 3: the concerted migration of two Na^+ ions within the *ab* plane, which related to the Na_e-Na_e-Na_e. Path 4: the migration of one Na^+ ions within the *ab* plane, correspond to the Na ion migration in the chain of Na_f. (Na_f-Na_f) Path 6: the concerted migration of two Na^+ ions within the *ab* plane, where Path 6 is closer to the Ni in TM layer. This path related to the Na_e-Na_e-Na_e.

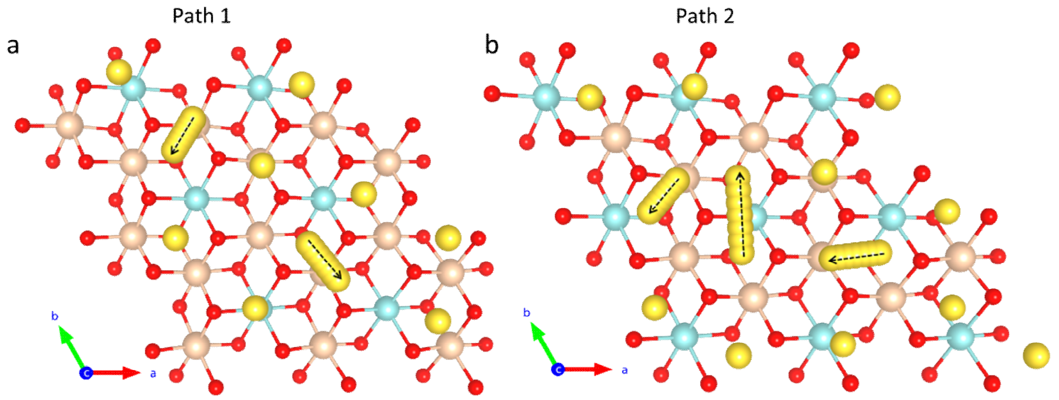

Figure S20. Na-ion diffusion in P2- $Na_{2/3}Ni_{1/3}Mn_{2/3}O_2$ of LZZ type ordering ($2[\sqrt{3}a \times \sqrt{3}b \times 1c]R30°$-type supercell) with monovacancy. The illustration of (a) Paths 1 and (b) 2 in P2-$Na_{2/3}Ni_{1/3}Mn_{2/3}O_2$ atomic structure. Path1 :the concerted migration of two Na^+ ions within the *ab* plane, which related to the Na_e-Na_f-Na_e. Path 2: the concerted migration of three Na^+ ions within the *ab* plane, which related to the Na_e-Na_f-Na_e and Na_e-Na_e.

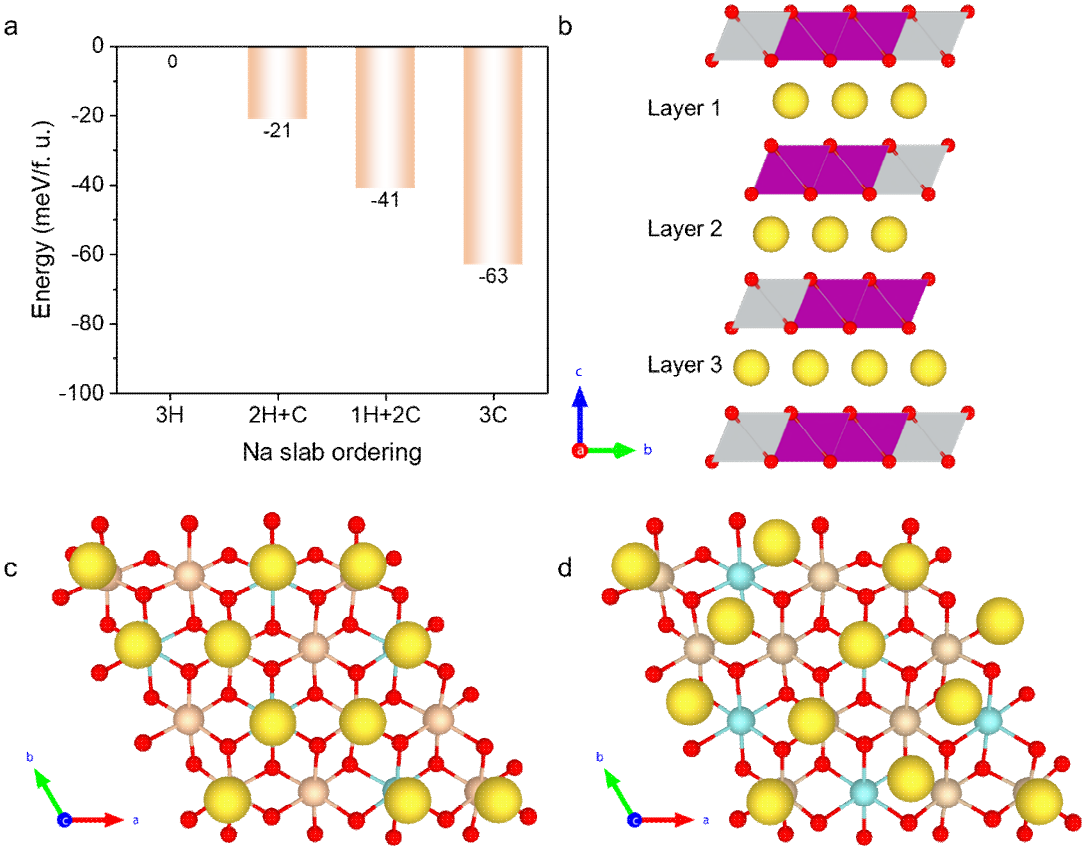

Figure S21. (a) Formation energies of different Na ion occupations in P3- $Na_{2/3}Ni_{1/3}Mn_{2/3}O_2$ structure, where the P3-type supercell is constructed using a $3a \times 3b \times 1c$-type lattice with 99 atoms ($Na_{18}Ni_9Mn_{18}O_{54}$). The 3H means three layers in honeycomb ordering, the 2H+C corresponds to two layers in honeycomb and one layer in chain orderings, the1H+C stands for one layer in honeycomb and two layers in chain orderings, and the 3C is all three layers in chain ordering. The simulation results indicate the chain type ordering is energy favourable structure. (b) Crystal environments of NaO_6 prism in P3 phases within the transition metal slabs. Atomic structure of P3-$Na_{2/3}Ni_{1/3}Mn_{2/3}O_2$ with the Na slab (c) honeycomb and (d) chain orderings.

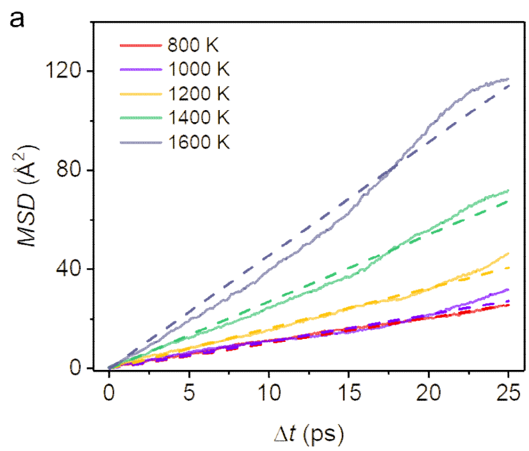

Figure S22. MSD of Na^+ ions in (a) P2-$Na_{2/3}Ni_{1/3}Mn_{2/3}O_2$ (LZZ ordering) and (b) P3-$Na_{2/3}Ni_{1/3}Mn_{2/3}O_2$ (chain ordering) as a function of time at different temperatures.

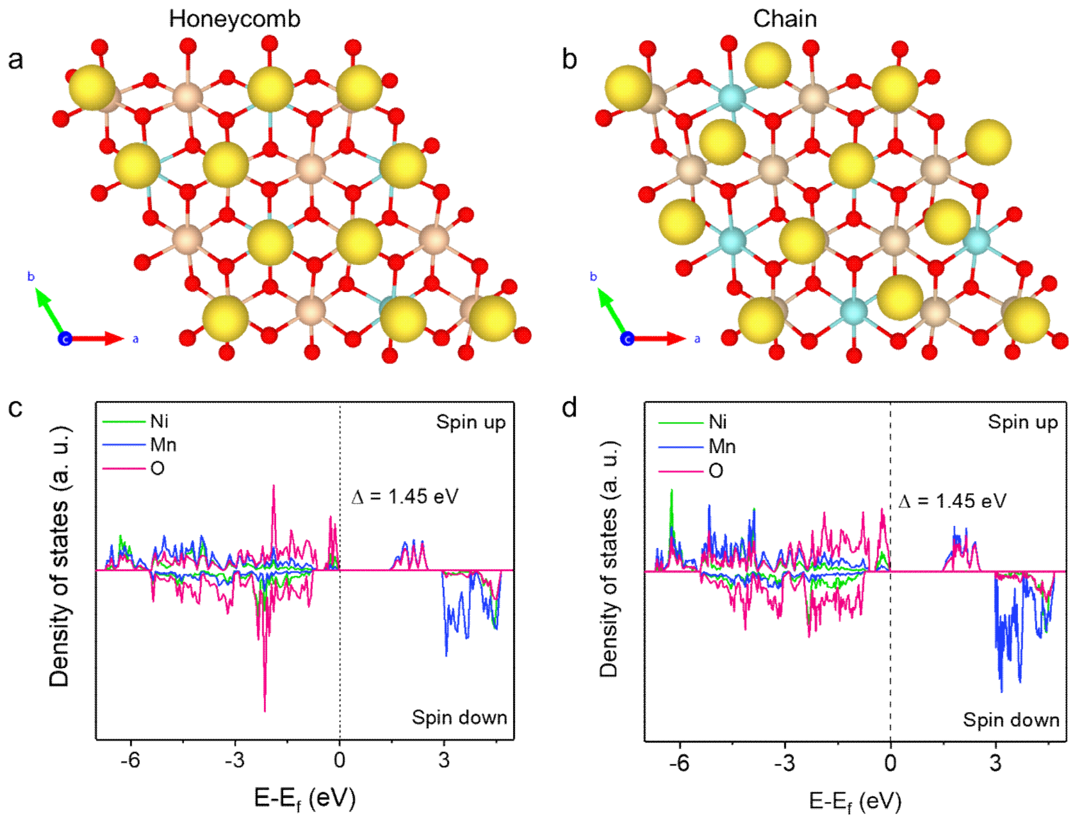

Figure S23. Atomic structure of P3-$Na_{2/3}Ni_{1/3}Mn_{2/3}O_2$ with (a) Na slab honeycomb ordering and (b) chain ordering. (c) Atomic projection of the density of states of (c) P3-$Na_{2/3}Ni_{1/3}Mn_{2/3}O_2$ (three-layer honeycomb ordering) and (d) P3-$Na_{2/3}Ni_{1/3}Mn_{2/3}O_2$ (three-layer chain ordering) from which direct charge tranfer gap was indentified. Simulation show that these two different sodium ordering structures have equal band gaps.

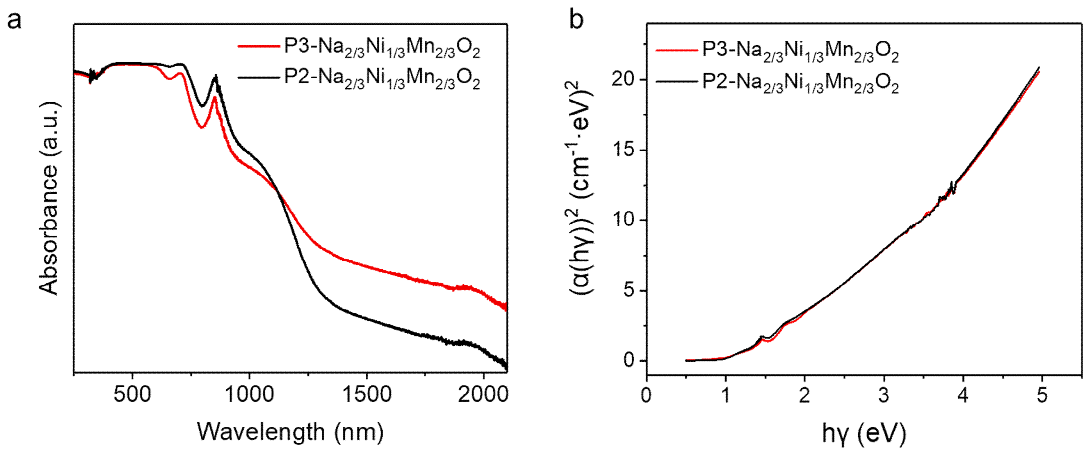

Figure S24. (a) Experimental ultra-visible light absorption spectra of P2-$Na_{2/3}Ni_{1/3}Mn_{2/3}O_2$ and P3-$Na_{2/3}Ni_{1/3}Mn_{2/3}O_2$ with the wavelength. (b) Dependence of $(\alpha h\nu)^2$ *vs.* photon energy ($h\nu$), from which the optical band gap is derived, above results indicate optic band gap of P2-$Na_{2/3}Ni_{1/3}Mn_{2/3}O_2$ and P3-$Na_{2/3}Ni_{1/3}Mn_{2/3}O_2$ is equivalent roughly.

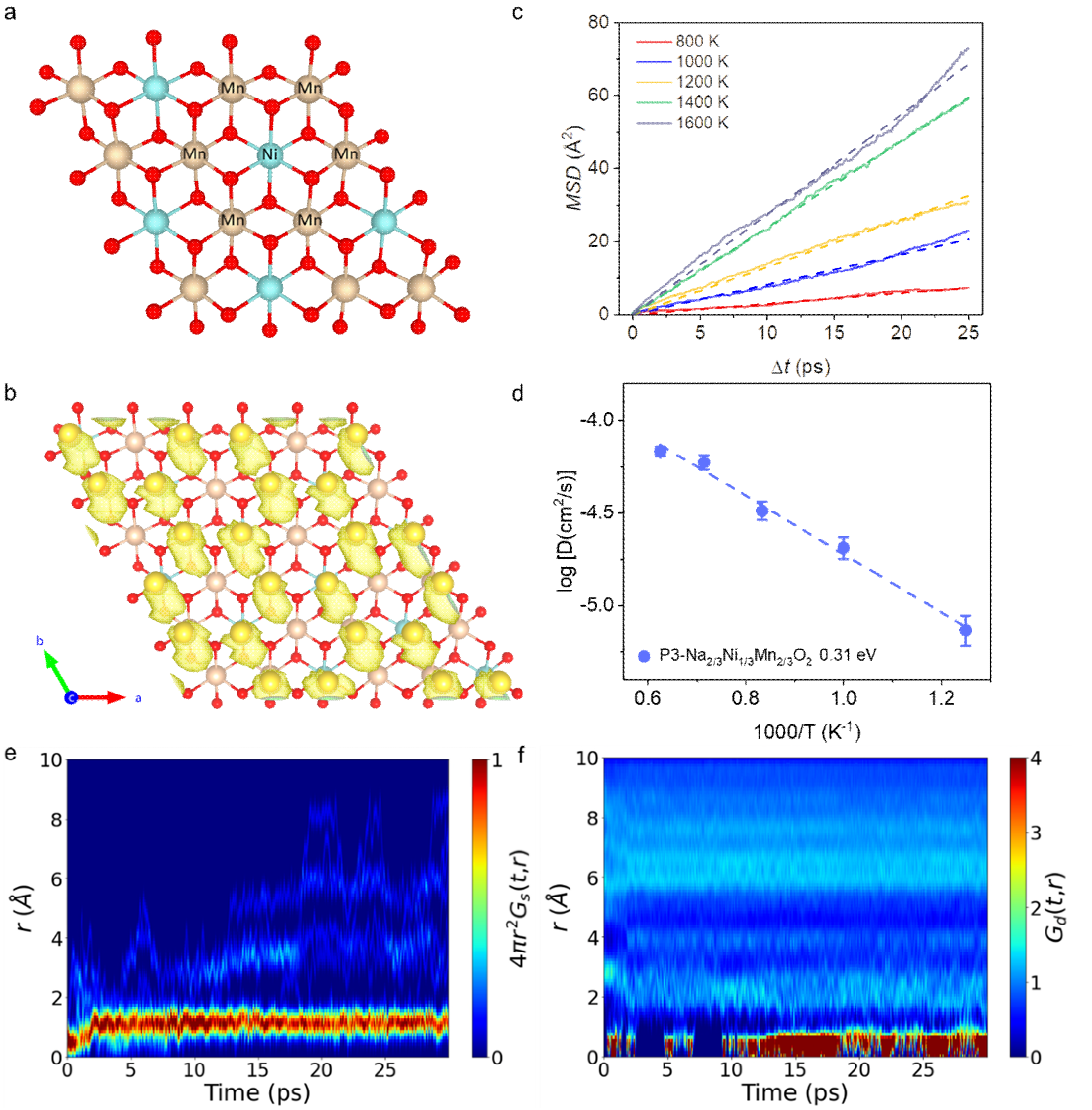

Figure S25. (a) Transition metal plane with the honeycomb ordering of Mn^{4+} and Ni^{2+}ions in P3-$Na_{2/3}Ni_{1/3}Mn_{2/3}O_2$. (b) The Na^+-ion diffusion pathway in P3- $Na_{2/3}Ni_{1/3}Mn_{2/3}O_2$ (Na slab honeycomb ordering) within the ab plane from AIMD at 800 K, and the scalebar of the isosurface is set to 0.001. (c)MSD of Na^+ ions in P3 $Na_{2/3}Ni_{1/3}Mn_{2/3}O_2$ (honeycomb ordering) as a function of time at different temperatures. (d) Arrhenius plot of Na^+ ion diffusivity in P3-$Na_{2/3}Ni_{1/3}Mn_{2/3}O_2$ (honeycomb ordering) from AIMD simulations. The error bars are the standard deviation of linear fit of MSD–Δt curves. (e) The self-part of the van Hove correlation function (G_s) for sodium in P3-$Na_{2/3}Ni_{1/3}Mn_{2/3}O_2$. (f) The distinct part of the van Hove correlation function (G_d) for sodium ions in P3-$Na_{2/3}Ni_{1/3}Mn_{2/3}O_2$. Both G_d and G_s are functions of the average Na−Na pair distance (**r**) and time step after thermal equilibration at 800 K.

Supplementary Note 5.

As for the stable thermodynamic configuration, from a formation energy point of view, the van Hove correlation function and probability density in P3-$Na_{2/3}Ni_{1/3}Mn_{2/3}O_2$ (chain ordering) from AIMD simulations is presented in Figure 5, and the van Hove correlation function and probability density in P3-$Na_{2/3}Ni_{1/3}Mn_{2/3}O_2$ (honeycomb ordering) from AIMD simulations is provided in Figure S25. The P3-$Na_{2/3}Ni_{1/3}Mn_{2/3}O_2$ (honeycomb ordering) exhibits a much higher activation energy of 0.31 eV. No connected channels are available within the Na slabs and the it seems difficult enough for one Na^+ ion diffusion to adjacent sites as shown in Figure S25 b. the peak in G_s between 0 and 2.0 Å remains unchanged after the thermal equilibration process, which implies that the Na^+ ions have a higher probability of staying at the initial position and are difficult to diffuse away to the neighboring sites in P3-$Na_{2/3}Ni_{1/3}Mn_{2/3}O_2$ as shown in Figure S25 e. The observed time scale of correlation (G_d) in P3-$Na_{2/3}Ni_{1/3}Mn_{2/3}O_2$ is around ~10 picoseconds at 800 K, which is much longer than that in P2-$Na_{2/3}Ni_{1/3}Mn_{2/3}O_2$ (~5 picoseconds). the van Hove correlation function indicates that Na^+ in P3-$Na_{2/3}Ni_{1/3}Mn_{2/3}O_2$ has a much higher probability of returning to their initial positions and staying there for a longer time duration than in P2-$Na_{2/3}Ni_{1/3}Mn_{2/3}O_2$.

Of particular importance is that much lower activation energy is observed than in P2-$Na_{2/3}Ni_{1/3}Mn_{2/3}O_2$ with respect to that in both chain and honeycomb ordering P3-$Na_{2/3}Ni_{1/3}Mn_{2/3}O_2$. Hence, the AIMD results support the conclusion fully.

Supplementary Tables

Table S1. Rietveld refinement results of XRD data for the P2-$Na_{2/3}Ni_{1/3}Mn_{2/3}O_2$ material.

	Overall Composition: P2-$Na_{2/3}Ni_{1/3}Mn_{2/3}O_2$ Space Group: *P*3. No. 143					
elem.	mult.	x	y	z	frac.	Uiso
Na	3	0.16667	0.66666	0.25	1	0.015216
Na	3	0.66667	0.5	0.75	1	0.015216
Na	3	0.33333	0	0.25	1	0.015216
Mn	3	0	0.5	0	1	0.0011
Mn	3	-0.33333	-0.16667	0.5	1	0.00108
Mn	3	0.33333	0.16667	0.5	1	0.00108
Mn	3	-0.33333	-0.16667	0	1	0.00108
Ni	3	0.33333	0.16667	0	1	0.00108
Ni	3	0	0.5	0.5	1	0.00108
O	3	0.66667	0	0.0872	1	0.0065
O	3	-0.16667	0	0.9128	1	0.006455
O	3	0.5	0.33333	0.9128	1	0.006455
O	3	0.16667	0.66666	0.9128	1	0.006455
O	3	-0.16667	0	0.5872	1	0.006455
O	3	0.5	0.33333	0.5872	1	0.006455
O	3	0.16667	0.66666	0.5872	1	0.006455
O	3	0.66667	0	0.4128	1	0.006455
O	3	0.33333	-0.16667	0.0872	1	0.006455
O	3	0	0.16666	0.0872	1	0.006455
O	3	0.66667	0.5	0.0872	1	0.006455
O	3	0.33333	-0.16667	0.4128	1	0.006455
O	3	0	0.16666	0.4128	1	0.006455
O	3	0.66667	0.5	0.4128	1	0.006455
O	3	0.33333	0	0.9128	1	0.006455
O	3	0.33333	0	0.5872	1	0.006455
Na	1	0	0	0.25	1	0.015216
Na	1	0	0	0.75	1	0.015216
Mn	1	0	0	1	1	0.00108
Ni	1	0	0	0.5	1	0.00108
Na	1	0.66667	0.33333	0.25	1	0.015216
Mn	1	0.66667	0.33333	0.5	1	0.00108
Mn	1	0.66667	0.33333	0	1	0.00108
Na	1	0.33333	0.66667	0.75	1	0.015216
Mn	1	0.33333	0.66667	0.5	1	0.00108
Ni	1	0.33333	0.66667	1	1	0.00108

Lattice constants: a = b = 10.00565 Å, c= 11.15304 Å, V = 966.972 $Å^3$.

Table S2. Rietveld refinement results of NPD data for the P2-$Na_{2/3}Ni_{1/3}Mn_{2/3}O_2$ material.

	Overall Composition: P2-$Na_{2/3}Ni_{1/3}Mn_{2/3}O_2$ Space Group: *P*$6_3$22. No. 182					
atom	site	x	y	z	frac	U_{iso}
O	12i	1.97909	0.31136	-0.15697	1	0.00249
Na	6g	2	0.36284	0.5	0.500	0.02969
Na	2a	0	0	0	0.0833	0.02969
Na	4f	1.33333	0.66667	0.00397	0.0833	0.02969
Mn	2b	0	0	-0.25	1	0.00694
Ni	2d	1.33333	0.66667	-0.25	1	0.00694
Mn	2c	0.66667	1.33333	-0.25	1	0.00694

Lattice constants: a = b = 4.93066 Å, c= 10.99454 Å, V = 231.482 $Å^3$.

Table S3. Rietveld refinement results of XRD data for the P2-$Na_{2/3}Ni_{0.3}Mn_{0.7}O_2$ material.

		Overall Composition: P2-$Na_{2/3}Ni_{0.3}Mn_{0.7}O_2$ Space Group: $P6_3/mmc$. No. 194				
atom	site	x	y	z	frac	U_{iso}
O	4f	0.3333	0.6667	0.0872	1	0.048
Na	2d	0.3333	0.6667	0.75	0.52	0.053
Ni	2a	0	0	0	0.3	0.0089
Mn	2a	0	0	0	0.7	0.0089
Na	2b	0	0	0.25	0.146	0.053

Lattice constants: a = b = 2.882344 Å, c= 11.162893 Å, V = 80.315 Å^3.

Table S4. Rietveld refinement results of NPD data for the P2-$Na_{2/3}Ni_{0.3}Mn_{0.7}O_2$ material.

	Overall Composition: P2-$Na_{2/3}Ni_{0.3}Mn_{0.7}O_2$ Space Group: *P*$6_3$22. No. 182					
atom	site	x	y	z	frac	U_{iso}
O	12i	1.97909	0.3117	-0.15688	1	0.00244
Na	6g	2	0.3499	0.5	0.566	0.0315
Na	2a	0	0	0	0.05	0.0315
Na	4f	1.33333	0.66667	0.0007	0.05	0.0315
Mn	2b	0	0	-0.25	1	0.0077
Ni	2d	1.33333	0.66667	-0.25	1	0.0077
Mn	2c	0.66667	1.33333	-0.25	1	0.0077

[a]Lattice constants: a = b = 4.998542 Å, c= 11.164374 Å, V = 241.575 $Å^3$.

Table S5. Fitting results of the different peaks of CV curves for P2-$Na_{2/3}Ni_{1/3}Mn_{2/3}O_2$, P2-$Na_{2/3}Ni_{0.3}Mn_{0.7}O_2$ and P2-$Na_{2/3}Al_{1/24}Ni_{7/24}Mn_{2/3}O_2$ electrodes.

Voltage range	Anodic peak (slope)	P2- $Na_{2/3}Ni_{1/3}Mn_{2/3}O_2$
2.0-4.0 V	a	0.04235
	b	0.06142
	Anodic peak (slope)	P2- $Na_{2/3}Ni_{0.3}Mn_{0.7}O_2$
	b'	0.02057
	b'	0.03458
	Anodic peak (slope)	P2- $Na_{2/3}Al_{1/24}Ni_{7/24}Mn_{2/3}O_2$
	c	0.02646
	d	0.0436

Table S6. Fitting results of the different peaks of CV curves for P2-$Na_{2/3}Ni_{1/3}Mn_{2/3}O_2$ and P3-$Na_{2/3}Ni_{1/3}Mn_{2/3}O_2$ electrodes.

Voltage range	Anodic peak (slope)	P2-$Na_{2/3}Ni_{1/3}Mn_{2/3}O_2$	Anodic peak (slope)	P3-$Na_{2/3}Ni_{1/3}Mn_{2/3}O_2$
2.0-4.0 V	a	0.04235	a''	0.02829
	b	0.06142	b''	0.03581
2.0-4.3 V	a	0.01792	a''	0.01157
	b	0.02575	b''	0.01502
